# Inferior vena cava septic thrombosis due to gut perforation after accidental toothpick ingestion

**DOI:** 10.1259/bjrcr.20150522

**Published:** 2016-06-29

**Authors:** Dario Poretti, Lorenzo Carlo Pescatori, Giovanni Mauri, Luca Maria Sconfienza, Giorgio Brambilla

**Affiliations:** ^1^Department of Radiology, Istituto Clinico Humanitas, Milan, Italy; ^2^Postgraduation School in Radiodiagnostics, Università degli Studi di Milano, Milan, Italy; ^3^Department of Interventional Radiology, Istituto Europeo di Oncologia, Milan, Italy; ^4^Department of Diagnostic and Interventional Radiology, IRCCS Istituto Ortopedico Galeazzi, Milan, Italy; ^5^Department of Biomedical Sciences for Health, Università degli Studi di Milano, Milan, Italy

## Abstract

A 57-year-old male patient was referred to our emergency department complaining of irremediable abdominal pain associated with mild fever. Abdominal CT scan revealed the presence of a small bowel perforation caused by an ingested toothpick, in association with a subsequent inferior vena cava thrombosis.

## Clinical presentation

A 57-year-old male patient, with unremarkable previous history, reported to his physician for subtle onset of abdominal pain in the lower quadrants. The patient underwent ultrasound examination of the abdomen, which did not reveal any abnormality. After several days, the patient developed mild fever (37.8°C) and increased pain in the lower quadrants and was then referred to our emergency department. Physical examination demonstrated elective pain in the mesogastrium that was associated with lower extremity oedema. Blood test revealed increased levels of C-reactive protein, erythrocyte sedimentation rate and white blood cell count. As a first diagnostic step, abdominal radiography was performed, which was unremarkable.

## Diagnosis

**Figure 1.**
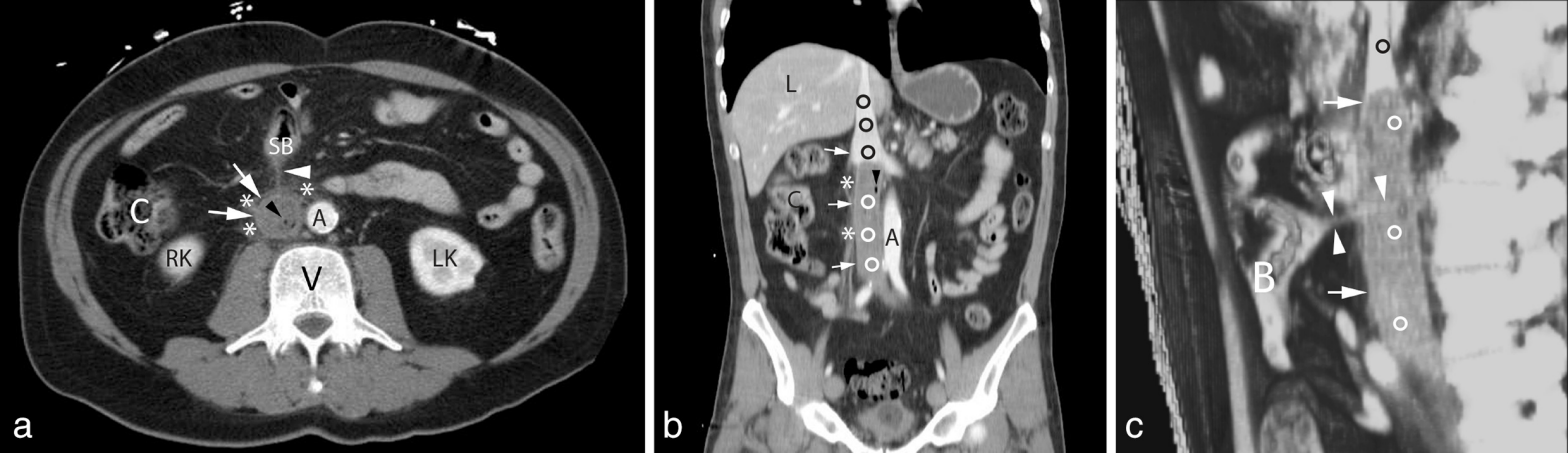
Contrast-enhanced CT scan of the abdomen showing small bowel perforation and inferior vena cava septic thrombosis caused by an accidentally ingested toothpick. (**a**) Axial image shows a complete thrombosis of the inferior vena cava (arrows) with some air bubbles within (black arrowhead). Perivascular phlogistic fat thickening can also be seen (asterisks). The toothpick can be appreciated as a thin linear image (white arrowhead) connecting the inferior vena cava and a small bowel loop (SB). (**b**) Coronal reformatted image confirms that the inferior vena cava (arrows) is partially occupied by a septic thrombus (white circles), containing a small air bubble (black arrowhead), surrounded by perivascular phlogistic fat thickening (asterisks). Note that the proximal inferior vena cava is patent (black circles). (**c**) A maximum intensity projection sagittal reformat shows the toothpick (white arrowheads) connecting a small bowel loop (B) with the inferior vena cava, in which a thrombus is present (white circles). Perivascular fat is thickened due to phlogosis (arrows). Note that the proximal inferior vena cava is patent (black circle). A, aorta; C, colon; L, liver; LK, left kidney; RK, right kidney; V, vertebral body.

A contrast-enhanced CT scan (6-slice; Brilliance, Philips, Eindhoven, Netherlands) was then performed, demonstrating the presence of an extensive thrombosis of the femoroiliac axis and the inferior vena cava up to the renal veins. Small air bubbles were demonstrated within the thrombus, associated with perivascular inflammatory reaction (Figure 1). These findings suggested the diagnosis of an infected thrombus. No free air or fluid collections, as well as abnormalities of solid organs of the abdomen were detected. A deeper analysis of the CT images allowed for recognizing the presence of a very thin low-density foreign body between the inferior vena cava, perforating an ileal loop and abutting straight into the vena cava itself. The object was correctly identified as a toothpick.

The patient was then sent to the operating theatre, where the foreign body was extracted first from the intestine and then from the vena cava. Examination during surgery revealed the presence of a toothpick that had perforated a small bowel loop and reached the inferior vena cava, thus causing the occurrence of a complete septic thrombosis. The patient also underwent thrombolysis and antibiotic treatment, with full recovery.

## Discussion

Ingested foreign bodies are an uncommon, though well-known, cause of abdominal emergency. Specifically, toothpick perforation of the digestive tract is not a common condition, occurring in about 0.2/100,000 persons/year in the USA, with a mortality rate up to 18% due to misdiagnosis or clinical complications. Duodenum, distal ileum and sigmoid are the most frequent sites of perforation.^1,2^ In general, perforation tends to occur where angulations or changes of luminal calibre of the bowel are present.^1^

Moreover, migration of toothpicks to several other organs and structures, such as liver, gallbladder, aorta, iliac artery, ureter, urinary bladder and pleural space, has been reported.^3–6^

Most of the time, patients are unaware of foreign body ingestion; anamnestic recollection is therefore rarely useful in leading to the correct diagnosis.

Imaging evaluation has a low detection rate of toothpick as a foreign body, being able to detect toothpicks in only 14% of cases. As a consequence, symptoms can last for more than 6 months before a definitive diagnosis can be made, and usually the final diagnosis is made directly in the operating room.^6^

Nevertheless, our case reinforces the role of CT scan as a diagnostic work-up in the emergency department to make the correct diagnosis in non-traumatic abdominal emergencies. Thin section images and multiplanar reformatting, allowed by the use of a six-row multidetector CT scanner, made interpretation of images easier, despite the low density of toothpicks compared with other foreign bodies such as chicken and fish bones. However, inflammatory reaction surrounding the foreign body may make it appear as a hyperdense, linear object, eventually surrounded by a thickened intestinal wall, fat infiltration, localized peritoneum and intra-abdominal abscess, if a perforation occurs.^7^

When the perforation is symptomatic, as in our case, surgical removal of the toothpick, in addition to bowel repair, is the treatment of choice. Nevertheless, other treatments have been proposed such as endoscopic removal of the foreign body, as well as percutaneous drainage of the related abscess.^7–9^

To the best of our knowledge, the case we report is the first one ever described of a perforation of an ileal loop by a toothpick, with direct fistulization to the inferior vena cava, lying approximately 4 cm from the involved ileal loop. As a consequence, the patient presented with an infected inferior vena cava thrombosis, which rarely can be caused by abdominal septic processes.^2,10^ Because of this peculiar presentation, open surgery was chosen to treat the patient, in order to remove the toothpick as well as repair the abdominal wall and the inferior vena cava.

In conclusion, even if rare, a perforation caused by a foreign body should always be suspected in an emergency setting such as the one described, in order to promptly handle the problem and better treat the patient.

## Learning points

1. Toothpick ingestion may result in perforation of the inferior vena cava.
2. CT scan with multiplanar reconstruction may help in detecting even minimal foreign bodies.
3. CT scan with multiplanar reconstruction improves the interpretation of small difficult findings.

## Consent

Informed consent was obtained.
